# Wasted Sympathy

**Published:** 1901-10

**Authors:** 


### WASTED SYMPATHY.

When the American Veterinary Medical Association at its annual meeting at Atlantic City, with the largest list of members in attendance which had ever yet convened, summarily expelled Claude D. Morris from membership, a few conservative members expressed their hesitation at the manner in which it was done, and argued that, while they recognized his dastardly acts, they believed he was entitled to a trial and ordinary procedure. In calm, philosophical reason they were right; but in the case of Claude D. Morris, who already had a reputation as a renegade and a sneak in his double dealings in New York State on the Veterinary Jury bill, the Tuberculosis bill, and other matters, his vent of personal spite against one man, which endangered the interests of the veterinary profession at large, had been so malicious that there was not more than a moment's hesitation in suspending the by-laws and casting him out.

Among those who argued most ardently for the law to take its usual course were some of his immediate associates from New York State, though not one offered an apology for him.

Claude D. Morris was summarily expelled. (See resolution, page 660, this Journal.)

It was announced at once that a formal and proper legal charge would be made against Claude D. Morris, who could then have a fair trial before the New York State Veterinary Medical Society, which would convene in annual session at Ithaca the next week.

The charges were made in legal form by Dr. Rush S. Huidekoper, the Secretary of the Committee on Army Legislation of the American Veterinary Medical Association. They were delivered to the President of the New York State Veterinary Medical Society on September 5th. (See charges, page 671, this Journal.)

When the New York State Veterinary Medical Society convened at Ithaca, N. Y., on September 10th the Executive Committee reported the following:

"Meeting of the Executive Committee of the New York State Veterinary Medical Society, convened at 10.30 A. M. in Dr. Law's study.

"Roll-call: Dr. Bell, President; Dr. Berns, Dr. Williams, Dr. Ackerman, Dr. Morris.

"President Bell offered a communication, signed by Dr. Rush S. Huidekoper, purporting to be charges against Claude D. Morris. Dr. Bell submitted the matter to the Committee. Dr. Williams moved that such charges were out of order, and therefore ought not to be received, by reason of the fact that Dr. Huidekoper is not a resident of the State, and therefore not a member of the Society under the constitution; carried.

"Supplementary meeting of the Executive Committee, called to order by Dr. Bell. Present: Drs. Berns, Williams, Bell, Ackerman, and Morris.

"The President presented charges against Secretary Morris, signed by Dr. T. G. Sherwood. Said charges were read in the presence of Dr. Morris, who was asked to withdraw during their discussion. Dr. Ackerman was elected Secretary *pro tem.*

"After consideration of the charges it was moved by Dr. Williams, seconded by Dr. Berns, that we recommend to the Society that the incoming Secretary notify Dr. Morris of the charges pending, and cite him to appear before the Association at the next regular meeting and show cause why the charges should not be sustained.

"Motion carried; adjourned.

"E. B. ACKERMAN,
"Secretary pro tem."

The report would be ludicrous if it were not so contemptible.

First. Dr. Williams gets the Committee, of which Claude D. Morris is a member, present, to hear him; that Dr. Rush S. Huidekoper—an old member of the Society, a former President of the Society, a member in good standing, with dues paid up to date of meeting, a legally registered veterinarian in New York State—is not a member because his large luggage happens to be at the moment out of New York State. A half-hour later the Society elected new members indorsed by Dr. Huidekoper as a member.

Second. Following this subterfuge, when Dr. Thomas G. Sherwood promptly duplicated the charges, the Committee, having evaded the prior charges of Dr. Huidekoper, claimed that Claude D. Morris was entitled to a year's time—the next regular meeting—to have a hearing.

Will the gentlemen who at Atlantic City asked for a simple legal trial now have any sympathy for the Claude D. Morris whose intimates, under the ægis of a localized coterie, received the charges of September 5th and hid them until they could be delayed for a year? for the Claude D. Morris, Secretary of the New York State Veterinary Medical Society, who, knowing that he had been expelled from the American Veterinary Medical Association, and that charges would be preferred against him at Ithaca, failed to bring the minutes and records of the Society to the annual meeting for use of the Society and to deliver to his successor? and for the Claude D. Morris who sat in committee and as Secretary of the Society meeting heard the charges made against him, and never asked a hearing or a chance for public defence of himself?

The New York State Veterinary Medical Society indorsed without a dissenting voice the resolution of the American Veterinary Medical Association in regard to continuing the Army Legislation work. (See resolution, page 670, this JOURNAL.)

